# Impacts and mechanisms of metabolic reprogramming of tumor microenvironment for immunotherapy in gastric cancer

**DOI:** 10.1038/s41419-022-04821-w

**Published:** 2022-04-20

**Authors:** Lin Zhao, Yuanyuan Liu, Simiao Zhang, Lingyu Wei, Hongbing Cheng, Jinsheng Wang, Jia Wang

**Affiliations:** 1 The First Clinical College, Changzhi Medical College, Changzhi, Shanxi 046000 China; 2 Collaborative Innovation Center for Aging Mechanism Research and Transformation, Center for Healthy Aging, Changzhi Medical College, Changzhi, Shanxi 046000 China; 3 Key Laboratory of Esophageal Cancer Basic Research and Clinical Transformation, Heping Hospital Affiliated to Changzhi Medical College, Changzhi, Shanxi 046000 China; 4 Department of Microbiology, Changzhi Medical College, Changzhi, Shanxi 046000 China; 5 Department of Immunology, Center for Healthy Aging, Changzhi Medical College, Changzhi, Shanxi 046000 China

**Keywords:** Cancer, Mechanisms of disease

## Abstract

Metabolic disorders and abnormal immune function changes occur in tumor tissues and cells to varying degrees. There is increasing evidence that reprogrammed energy metabolism contributes to the development of tumor suppressive immune microenvironment and influences the course of gastric cancer (GC). Current studies have found that tumor microenvironment (TME) also has important clinicopathological significance in predicting prognosis and therapeutic efficacy. Novel approaches targeting TME therapy, such as immune checkpoint blockade (ICB), metabolic inhibitors and key enzymes of immune metabolism, have been involved in the treatment of GC. However, the interaction between GC cells metabolism and immune metabolism and how to make better use of these immunotherapy methods in the complex TME in GC are still being explored. Here, we discuss how metabolic reprogramming of GC cells and immune cells involved in GC immune responses modulate anti-tumor immune responses, as well as the effects of gastrointestinal flora in TME and GC. It is also proposed how to enhance anti-tumor immune response by understanding the targeted metabolism of these metabolic reprogramming to provide direction for the treatment and prognosis of GC.

## Facts

1. The interaction between gastric cancer cells and immune cells leads to metabolic competition in TME, restricts the nutrients required by immune cells, and leads to microenvironmental acidosis, which causes immune cells to undergo metabolic reprogramming and affects the progress of gastric cancer.
2. Invasive CD8 + T cells in tumor microenvironment are usually dysfunctional and have unique epigenetic expression, which are the main factors affecting anti-tumor immunotherapy.
3. In the tumor microenvironment, tumor cells produce a variety of metabolic wastes, such as lactic acid and high potassium ion concentration, which will change the tumor microenvironment and thus affect the tumor progression.
4. Infection with Helicobacter pylori will increase glycolysis, affect amino acid metabolism and increase toxic metabolites, all of which contribute to the progress of gastric cancer to a large extent.

## Open questions

1. How to target metabolic therapeutic targets based on tumor microenvironment, and what other effects do these therapeutic targets have on human body in addition to antitumor effects?
2. Immunotherapy has different effects on the classification of different cold and hot tumors. The curative effect on hot tumors is better than that of cold tumors. What means can be used to turn cold tumors into hot tumors?
3. Will the disordered immune metabolism in the tumor microenvironment balance with the immune metabolism of the human body, so as to slow down the proliferation of the tumor? If so, would such a balance be a good treatment?
4. Do tumor cells affect the epigenetic modification (methylation) of T cells by regulating the metabolites in their microenvironment, so as to affect the function of T cells and promote the occurrence and development of tumors? If so, what is the regulation mechanism?

## Introduction

GC is the fifth most common cancer and the third most common cause of cancer death globally [1]. GC is a multifactorial disease, and both the environmental factors and the genetic factors play a role in its pathogenesis [2]. GC is a highly heterogeneous malignancy with great differences in histological and molecular pathology. In 2014, the Cancer Genome Atlas (TCGA) Research Network reported the atlas results based on the detection of the genomes of 295 primary gastric adenocarcinomas [3]. They established four genomic subtypes of gastric adenocarcinoma, including microsatellite instability (MSI + ) (22%), EBV + (9%), low genomic stability (GS) (20%), and high chromosomal instability (CIN) (50%) [4].The occurrence, development and metastasis of GC are inseparable from its survival environment -- TME.

The TME is an environment conducive to the growth and expansion of cancer cells. Tumors survive through immune metabolic reprogramming and altered immune cells that occur in TME including activating immune cells and stromal cells such as TAM [5], tumor-associated neutrophils (TAN) [6], Treg [7], MDSCs [8], ECs [9], CAF [10] to initiate metabolic reprogramming, by changing cell metabolism in the microenvironment such as increased “Warburg effect” [11], palmitoylation of proteins affecting oncogenes and tumor suppressor genes [12] and abnormal upregulation of fatty acid synthesis to promote the occurrence and development of tumors, or by competing for oxygen and glucose in the energy limited microenvironment [13], essential or non-essential amino acids [14, 15] and other nutrients to create an environment conducive to the growth of the tumor itself. Meanwhile, the TME in the stomach is unique. Now studies on metabolism of TME in GC are roughly expounded from two aspects: on the one hand, the metabolism of GC cells itself is showed to reveal the distinctive metabolic characteristics of GC cells compared with normal cells, so as to further intervene with inhibitors or other targeted drugs to achieve the purpose of treating GC. Another aspect is the metabolism of tumor-infiltrating immune cells. Since the special TME in GC affects the metabolism of immune cells, it is often studied in combination with the metabolic pattern of tumor cells. In TME, in addition to tumor cells themselves escaping immune system attack through metabolic reprogramming evolution, many immunosuppressive cells can also express many extracellular enzymes, such as IDO, CD73, ARG1, etc., depleting nutrients to promote tumor progression. At the same time, TME also produces many immunosuppressive metabolites, such as canine, adenosine and so on. Therefore, metabolic genes that regulate TME have been discovered continuously in recent years, and drugs based on these metabolic targets have shown good antitumor effects in some tumor models. Besides the above-mentioned tumor promoting pathways, the unique bacteria colonized in the stomach also affect the development of tumors. The unique TME has created an opportunity for immunotherapy.

Currently, the combination of neoadjuvant chemoradiotherapy, molecular targeted therapy and immunotherapy is the main treatment for advanced GC [16]. Among them, immunotherapy has attracted much attention, and the survival environment of tumor—TME has to be mentioned when referring to immunotherapy. A growing body of evidence illustrates the clinicopathological significance of changes in TME in predicting prognosis and therapeutic efficacy [17]. Immunotherapy with checkpoint inhibitors is a new treatment approach rapidly entering clinical practice for malignant melanoma and renal cell carcinoma [18]. Although targeted therapy has emerged, the effect of targeted therapy for gastric cancer is not particularly prominent. Immunotherapy related to immune metabolic reprogramming in the tumor microenvironment is not perfect, so we systematically summarize the possible therapeutic targets of immune metabolic reprogramming and how to use immunotherapy targets to enhance anti-tumor immunity of gastric cancer by systematically summarizing the metabolic characteristics of immune cells in the tumor microenvironment.

So, we discuss the role of immune metabolism in TME and the progression of GC based on such a special environment of gastric microflora, and to propose corresponding therapeutic targets based on TME in GC and its mechanism of action. A comprehensive description of the characteristics of TME in GC can help to explain the response of GC related to immunotherapy and provide new strategies for cancer treatment [17].

### Microenvironment of GC

TME is the battlefield between tumor cells and anti-tumor cells, and the TME in GC is unique. TME houses a variety of immune cells, including helper T (Th) cells, regulatory T (T_reg_) cells, dendritic cells (DCs), tumour-associated macrophages (TAMs), mesenchymal stem cells (MSCs), and associated inflammatory pathways, which have been reported in GC cases [17, 19, 20]. These stromal cells promote tumor growth by releasing various molecules that directly activate cancer cell growth signals or reshape surrounding areas [21]. Among the numerous tumor promotion pathways, the unique flora colonized in stomach also affect the development of tumors. The change of microflora is important for the progression of GC. In normal stomach, helicobacter pylori (H. pylori) negative individuals have highly diversified gastric microbiota, which is dominated by five phyla: Proteobacteria, Firmicutes, Actinobacteria, Bacteroidetes and Fusobacteria [22]. However, compared with the microflora of normal stomach, the microflora of GC patients was dominated by Lactobacillus, Streptococcus, Virococcus, Prevococcus and H. pylori [23]. Among them, H. pylori, the classic strain of GC, has profound significance for the initiation of cancer. For instance, H. pylori can inject virulence factors CagA and lipopolysaccharide (LPS) into gastric epithelial cells to drive the development of GC [24, 25]. H. pylori can affect gastric epithelial cells in many ways, such as DNA damage, apoptosis, proliferation, stimulation of cytokine production and promotion of cell transformation [26]. In the presence of large numbers of H. pylori, active inflammation caused by innate immune disorders in the TME promotes carcinogenesis and postoperative recurrence [27]. During this process, a large number of cell metabolic by-products such as lactic acid [28], adenosine [29], nitric oxide (NO) [30], potassium ion (K^+^) [31] and reactive oxygen species [32] accumulate in the microenvironment, resulting in abnormal pH and oxygen levels in the TME. Thus, the components of the TME tend to complicate and worsen. In this case, the function of immune cells recruited into the microenvironment is limited [33], and the accumulation of lactic acid makes the immune cells more tolerant, thus promoting tumor and angiogenesis [34, 35].

Furthermore, the immune cells of the body also carry out immune resistance to the tumor cells in the TME. DCs [36], effector T(T_eff_) cells [37], memory T cells (T_mem_) cells [38], and natural killer (NK) cells [39] are activated to control the tumor and prevent immune evasion and disease progression. These immune cells fight tumor cells by exhibiting properties that are contrary to the metabolites that cause them to fluctuate in the microenvironment. Some cells even have a dual effect on tumors. Therefore, the dynamic changes of various metabolites in the TME guide the direction of tumor development.

**Fig. 1:**
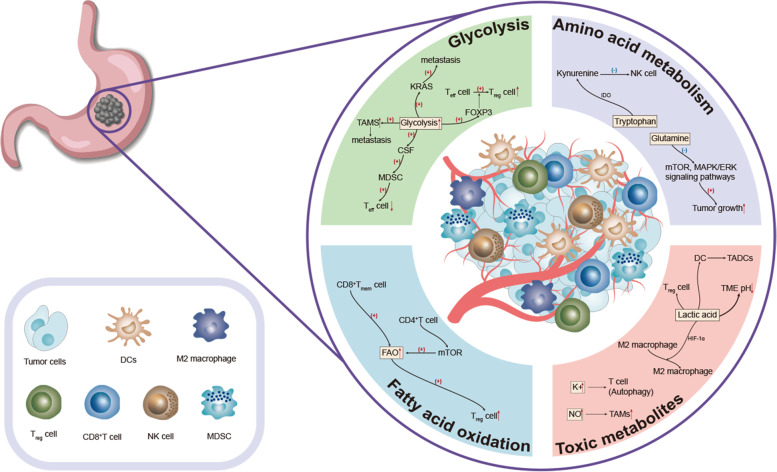
Overview of the TME. The TME is the environment that tumors depend on for survival and is often described as low oxygen, low pH, and various metabolic changes—enhanced glycolysis, abnormal amino acid and fatty acid metabolism. These changes also led to changes in immune cells in the microenvironment, with an increase in various tumor-related immune cells and a decrease in inhibitory cells—more TAMs, tumor-associated dendritic cells (TADCs), Treg and M2-like macrophages, fewer Teff cells, and more CD8^+^Tmem cells, and various toxic metabolites glutamine, canine urine, and lactic acid also increased.

In the stomach, these changes in the TME reveal to us the characteristics of tumor cells and anti-tumor cells in the microenvironment. In view of the characteristics of these microenvironments, specific selective inhibition of tumor cells and specific up-regulation or down-regulation of substances, to provide a suitable environment for anti-tumor cells to grow and play a role, that is, we are looking for a target for the treatment of GC. We summarize the characteristics of GC TME to provide therapeutic strategies for the treatment of GC (Fig. 1).

## Metabolic changes in the microenvironment of GC

### Glycolysis

In 1956, Otto Warburg first observed that tumor cells, compared to non-tumor cells, typically undergo glycolysis rather than oxidative phosphorylation (OXPHOS) for energy. This metabolic phenomenon is known as aerobic glycolysis or “Warburg effect” [40, 41]. The enhanced glycolytic activity of cancer cells and poor blood exchange are important reasons for the limited availability of glucose in TME [42]. This enhanced glycolysis and aerobic metabolism of pyruvate to lactic acid rather than OXPHOS to produce energy are closely associated with the initiation, development, invasion, metastasis, drug resistance, and poor prognosis of GC [43].

Glucose is the basic raw material in glycolysis.Through co-culture of lymphoma cells and T cells, it is found that there is competition between them for nutrients such as glucose, which limits their ability to produce effector cytokines [44]. Thus, glucose is not only the primary fuel source for activating T cells, but also ensures their persistence when the adaptive immune response is initiated [45]. Through experiments in which T cells were infiltrated in mouse and human tumors, the experimenters observed the loss of tumor-infiltrating T cells mitochondria and their dependence on glycolytic metabolism, resulting in the inability of these T cells to perform key cell function in the low-sugar TME [46]. At the same time, accelerated glycolysis metabolism in cancer cells will increase colony-stimulating factor (CSF) and macrophage CSF to promote MDSC infiltration and further inhibit T_eff_ cell function [47]. In addition, low glucose induced FoxP3 expression, thereby increasing the differentiation of T_eff_ cells into T_reg_ cells [48]. The T_reg_ transcription factor FoxP3 reprograms T cell metabolism by inhibiting glycolysis and enhancing OXPHOS. This metabolic change gives T_reg_ cells a metabolic advantage in a low glucose and lactic acid environment [49]. By simulating TME with high and low glycolysis in vitro, we found that the effect of CTLA-4 (involved in the down-regulation of T cell glycolysis) blocking the promotion of T_reg_ cells instability was dependent on T_reg_ cells glycolysis and CD28 signaling [50]. These results indicate that low degree of glycolysis is conducive to maintaining the tumor-promoting effect of T_reg_ cells. In clinical practice, the distribution of T_reg_ cells in patients with GC was studied, and the relationship between T_reg_ cell imbalance and the clinic was evaluated. It was found that as the disease progressed, the accumulation of T_reg_ cells in the TME gradually increased, leading to an imbalance of T_reg_ cells in patients with GC. In addition, T_reg_ cells promote tumor progression by secreting transforming growth factor-β (TGF-β) to help cancer cells escape from host immune surveillance [51].

In addition to blocking the immune system, the TME that changes during the progression of certain tumors can instigate the conversion of macrophages to TAMs [52]. TAMs is becoming a key player in GC development [53]. In a low glucose environment, TAMs re-regulate its own functions by activating glycolysis [5]. TAMs mainly provides nutrients to malignant cells by indirectly increasing the availability of selected nutrients in TME, and simultaneously activates powerful immunosuppressive function to support tumor progression [54]. The mechanism of TAMs’ nutritional support for malignant cells is the recruitment or activation of ECs through TAMs derived products, including the production of AMD (TAM-derived adrenomedullin) and chemokine (C-X-C motif) ligand 12 (CXCL12), to generate new angiogenesis [55, 56]. Because of its role in promoting cancer progression and immunosuppression, targeting TAM is also a promising target for cancer treatment. TAM works by binding to TAM receptors (Tyro3, Axl, and MerTK), a family of receptor tyrosine kinases that polarize macrophages in favor of tumor-like M2 phenotypes [57]. So how can tumor cells affect the body through M2 macrophages? The researchers found that compared with GC tissues sensitive to 5-FU, YAP1 (Yes associated protein 1) is overexpressed in resistant GC tissues. In addition, IL-3 secreted by YAP1 overexpressed GC can polarize macrophages into an M2-like phenotype and induce a GLUT3-dependent glycolysis program. At the same time, polarized M2 macrophages enhance the resistance of tumor cells to 5-FU (The antimetabolite 5-fluorouracil) by secreting CCL8 and activating the phosphorylation of JAK1/STAT3 signaling pathway [58].

By studying the transcriptomes of paired colorectal cancer cell lines with different KRAS gene mutation status, we found that glucose transporter-1(GLUT1) is continuously up-regulated in KRAS mutant cells, and mutant cells can survive under low glucose conditions. In contrast, cells with the wild type allele of KRAS were less likely to survive in the presence of low glucose. These data suggest that low glucose can mutate the KRAS pathway leading to human tumor initiation [59]. The researchers detected the activation of KRAS in the production of epithelial-mesenchymal transition (EMT) and cancer stem cell-like cells (CSC). It was found that the activation of KRAS in gastric adenocarcinoma cells stimulates EMT and transforms EMT into CSCs, thereby promoting tumor metastasis [60]. PKM2, a key catalyzed enzyme of glycolysis, is elevated in GC with a certain degree of clinical significance. The expression of PKM2 in human GC cell lines is higher than that in normal gastric mucosal EC lines. PKM2 knockdown can inhibit cancer cell metastasis by inhibiting E-cadherin and promoting the expression of vimentin and N-cadherin. It also inhibits the EMT phenotype, indicating that the high expression of PKM2 is associated with the poor survival rate of patients with GC and is related to its metastasis [61]. For this reason, we suspect that knocking down PKM2 may be important in slowing the progression of GC.

Obviously, because glucose is an important survival substance in competition between cells, the cells that play the tumor-promoting and anti-tumor effects in the TME will have greater efficacy if they have more glucose. Glycolysis causes the low glucose effect of TME, with the release of CSF, FoxP3 and TGF-β, etc., which changes the metabolism mode of immune cells, thereby creating a nutritious and less disturbing living environment for tumors. In addition, the low glucose in TME changes the differentiation direction of anti-tumor cells, leading to blindness of the immune inspection system and paralysis of the immune elimination system. In summary, the low-glucose environment in the TME is an environment conducive to the survival of tumor cells, which is beneficial to the development of tumor cells. With this feature, a treatment method that mainly improves the low glucose content in the TME can be proposed.

### Amino acid metabolism

For cell populations living in the TME, especially those in a highly proliferative state, amino acids are involved in the composition of the TME as essential resources and metabolites for cell survival. Among them, Mitogen-activated protein kinases (MAPKs) plays an important role in tumor development, and amino acids related to MAPKs classical pathway will affect tumor progression with changes in concentration.

Studies have shown that the nutrient arginine in the TME promotes activation of MAPKs pathways by blocking the dephosphorylation and inactivation of MAPK kinase tumor-promoting locus 2 (TPL-2) [62]. Several compelling body of evidences showed that arginine is involved in the metabolism of polyamines [63], proline [64], creatine [65] and other important components that have direct effects on TME. It is worth mentioning that arginine is also an important source of NO, which plays an important regulatory role in tumor development [66]. In addition, some tumors present argininosuccinatesynthase1 (ASS1) deficiency, where exogenous arginine in the TME is of great significance for the survival of these arginine-incapable tumor cells [67]. Arginine is critical for the activation of MAPK kinase (MEK). In the case of bacterial LPS stimulation of macrophages, arginine has the effect of activating ERK1/2. Arginine feeding to hungry mice promotes ERK1/2 activation and macrophage production of TNF-α [62].The resulting TNF-α researchers found that in the co-culture model of GC cells and cancer-associated fibroblasts (CAFs), GC cells release pro-inflammatory cytokine TNF-α through the TNFR2-NF-κB-IRF-1 pathway, which can induce CAFs to secrete IL-33, IL-33 derived from CAFs activates the ERK1/2-SP1-ZEB2 pathway in a st21-dependent manner to induce EMT, thereby enhancing the migration and invasion of GC [68].

In addition to being a raw material for the synthesis of arginine or other non-essential amino acids, purine, pyridine and glucose, glutamine is rapidly consumed to provide nutrients for rapidly proliferating cells and redox to remove reactive oxygen species, making glutamine the focus of TME studies [69, 70]. Experiments on pig intestinal ECs showed that glutamine deficiency inactivated mTOR and MAPK/ERK signaling pathways by interfering with amino acid metabolism in cells, and the pathways were reactivated after glutamine supplementation [71]. Glutamine is necessary for T cell function. The consumption of glutamine will inhibit the proliferation of T cells and the production of cytokines. At this time, even if the precursors of glutamine biosynthesis are increased, the inhibitory effect on T cells cannot be reversed [72]. Glutamine is also essential for cancer cells. In one study, researchers described the metabolic characteristics of glutamine using two cohort studies and concluded that high consumption of glutamine was associated with poor prognosis and survival in cancer patients [73]. Since glutamine is essential for both immune and tumor cells, research has followed. Researchers used qRT-PCR to detect the expression of CirB3GNTL1 and miR-598 in the stomach and cell lines and evaluated the expression of glutamine, glutamate, and α-ketoglutarate (α-KG). Experimental results showed that the expression of CircB3GNTL1 was up-regulated in GC tissues and cell lines, and the expression of miR-598 was up-regulated. Compared with normal paracarcinoma and GC cell lines, knocking down circB3GNTL1 can prevent GC cell proliferation and glutamine decomposition [74]. The treatment aimed at the circB3GNTL1 target may have a good effect on the treatment of GC.

Tryptophan is an essential amino acid in the body and plays an important role in cell metabolism in the microenvironment. Reduced concentrations of tryptophan in vivo and elevated levels of its metabolite kynurenine have been associated with the development of cancer [75]. Kynurenine interferes with the expression of NK cells and the function of NKp46 and NKG2D receptors, downregulates NK cells related cytokines, inhibits cancer immune surveillance and promotes immune escape [76]. As an endogenous ligand of aromatics receptor (AhR), kynurenine also regulates T_reg_ cells through AhR and inhibits Th17 differentiation [77, 78]. Several pieces of evidence demonstrated that inhibiting IDO will reduce Kyn/AhR-mediated T_reg_ differentiation, providing an effective strategy for the prevention and treatment of inflammation-related colon cancer, and has reference value for the treatment of GC [79]. IDO plays an important role in immunosuppressive pathways, and increased expression of IDO proteins can alter the number of Th1, Th17, Th22 and T_reg_ cells, which may lead to an increased risk of peptic ulcer development in patients infected with H. pylori, thereby increases the likelihood of GC [80]. Compared with other participating enzymes, indoleamine-2,3-dioxygenase 1 (IDO1) plays a decisive role in the decomposition of tryptophan into kynurenine [75, 81, 82]. Over expression of human IDO1 in a constitutive manner has been demonstrated in the clinicopathologic study related to GC [83] and an experiment using GC cell lines demonstrated that IDO1 and COL12A1 can promote lymphatic metastasis of GC through positive feedback regulation of MAPK pathway mediated by kynurenine and integral protein β1 [84]. Of note, overexpression of p38-MAPK/MSK1-mediated histone H3 serine 10 phosphorylation (H3S10ph) can be used to evaluate the prognostic value of negative resection range in GC [85]. Increased ph-MSK1 and PH-P38 levels in tumor tissues and decreased PH-MSK1 and H3S10ph levels in GC cells both confirmed that p38-MAPK /MSK1 pathway regulates H3S10ph in gastric cancer [85]. In addition, kynurenine inhibits T_eff_ cell function through direct and indirect pathways. For example, kynurenine can selectively induce thymocyte and Th1 cell apoptosis and/or induce immature CD4^+^T cells to differentiate into T_reg_ cells, reducing the number of T_eff_ cells [86, 87]. Furthermore, T_reg_ cells induced an increase of IDO1, and T_eff_ cells were further silenced in this positive feedback loop [78, 88].

To sum up, amino acids are an indispensable part of cell metabolism. For example, the increase of glutamine and arginine and the decrease of tryptophan and the increase of its metabolite kynurenine will promote the development of tumors. The characteristics of these amino acids influencing tumor progression also provide directions for related treatments. Multiple cytokines in tumor microenhractures such as TNF-α, IFN-γ, IL-6, and IL-10 play a synergistic role by increasing the expression of IDO1, affecting catabolism of tryptophan, and promoting immunosuppressive response, which in turn promotes the initiation of cancer [89, 90]. Similarly, arginase I in macrophages can be induced to weaken the immune capacity of mice [91], and arginase I can be induced by IL-6, IL-10 and CSF [92]. In general, glutamine, arginine and tryptophan can simultaneously regulate tumor progression and immune response. Whether it is the life activities of cancer cells or immune cells, such as the activation of naive T cells, the differentiation of immune cells, the formation of infiltrating blood vessels in tumors, and the proliferation of cancer cells, these high energy demand events all lead to high demand for amino acids in the entire microenvironment. A better understanding of amino acids and their use in the TME may help reveal new targets for GC immunomodulation.

### Fatty acid oxidation

By analyzing the metabolic reprogramming model of immune cells, we found that different immune cell groups had different processes of fatty acid metabolic pathways [93, 94]. In mouse melanoma model, CD8TILs enhances the signal and catabysis of PPAR- fatty acid to maintain its effective function, which is related to the low glucose level in TME, suggesting that fatty acid oxidation (FAO) may play an important role in some metabolically flexible immune cells in TME [95]. These metabolically flexible immune cells, which are primarily dependent on FAO rather than glycolysis, can survive in the low glucose environment of TME and exert their effect to promote tumor development.

The primary lipid metabolism of CD8^+^ T_mem_ cells is through FAO to support their survival and function. Mice with a T cell specific deficiency of TRAF6 showed a loss of antigen-specific cells within weeks of initial immunization. The reason is that the ability to produce T_mem_ cells is severely deficient due to the change in the expression of the gene regulating FAO, suggesting that FAO plays an important role in T_mem_ cells reactivation induced by repeat antigen [96]. Carnitine palmitoyl transferase 1 A (CPT1A) is a mitochondrial transporter that transfers long-chain fatty acids for FAO into mitochondria. Compared with T_eff_ cells, the continuous up-regulation of CPT1A expression in T_mem_ cells also proved that the survival of T_mem_ cells mainly depended on the FAO metabolic pathway. Preliminary studies using Etomoxir as a CPT1A inhibitor have shown that FAO is one of the main fuels for OXPHOS in T_mem_ cells [97]. Spare respiratory capacity (SRC) can indicate the extra ability of cells to produce energy in response to stress or activation, and can also be used as a parameter indicating the ability of cells to up-regulate OXPHOS. Relevant studies have shown that T_men_ cells have a higher spare respiration than other T cells [98, 99]. Thus, stronger SRC in T_mem_ cells promotes their survival in harsh conditions. This is also an important reason why T_mem_ cells can control tumors in the body for a long time [100]. In addition, the long chain FAO may be important for the immune cells residing in the tissues. The CD8^+^T cell subsets called tissue-resident memory cells specifically depend on fatty acid binding protein 4 (FABP4) and FABP5 to input extracellular fatty acids for FAO and maintain long-term memory phenotype. This characteristic allows immune cells to persist for a long time after the tumor disappears [101].

TAMs in TME will reprogram metabolism, and TAMs are mostly M2-like polarized. When TAMs tend to differentiate M2-like macrophages, it can promote the development of tumor cells, angiogenesis and immunosuppression. By inhibiting mitochondrial OXPHOS, specifically FAO, above evidence demonstrated that M2 macrophage development were also prevented. Therefore, M2 metabolism heavily favors the use of FAO and mitochondrial respiration to meet functional requirements. Fatty acid synthesis plays an important role in the differentiation and function of inflammatory macrophages by providing them with cell membranes and other key lipid cell structures [102, 103]. Derivatives of macrophages can also play a role in promoting tumors. Macrophage-derived S100A4 is the main source of extracellular S100A4, and S100A4 plays an important role in promoting tumor malignant development and mitochondrial metabolism [104, 105]. An experiment based on a mouse tumor model provided evidence for the mechanism by which TAM polarization toward protumor phenotypes, suggesting that macrophage S100A4 enhances m2-like polarization of proto-macrophages mediated by upregulation of peroxisome proliferator-activated receptor γ (PPAR-γ)-dependent FAO [106]. The increase in the number of M2-like macrophages in TME is associated with the decrease in the overall survival rate of several malignant tumors, including GC [107]. At the same time, the study also showed that the inhibition of FAO may limit the immunosuppression function of M2-like macrophages, which is of great significance for immunotherapy of GC [108, 109].

The researchers found that a subset of GC was characterized by high numbers of T_reg_ cells and low numbers of T_eff_ cells. Genomic analysis showed that mutations in RHOA in cancer cells (which promote tumor progression) activate the PI3K-AKT-mTOR signaling pathway, increasing the production of free fatty acids that are consumed more efficiently by T_reg_ cells than T_eff_ cells. Immune suppression from RHOA mutations is the basis for ICB resistance [110]. During the activation of CD4^+^T cells, FAO can be enhanced through the inhibition of mTOR, which is conducive to the production of T_reg_ cells [111]. Recently, it was found that the lipid transport protein CD36 can better adapt CD4^+^T_reg_ cells to TME by regulating mitochondrial fitness through peroxisome proliferator-activated receptor -β signaling, suggesting that the lipid transport protein CD36 is also necessary for CD4^+^ T_reg_ cells accumulation in TME.

Fatty acid metabolism reprogramming plays a key role in the occurrence and development of GC. Lipid biosynthesis pathway can be altered by changes in gene expression and enzyme expression levels and activities in various metabolic pathways. As previously mentioned, RHOA in cancer cells increases the production of free fatty acids better utilized by T_reg_ cells by activating the PI3K- Akt signaling pathway. FAO also occurs in different cells and has different effects on tumors.

T_mem_ cells increase FAO to maintain the immunity to tumor cells, and the cancer-promoting effect of FAO is reflected in TAMs polarization to M2 and T_reg_ cells proliferation.

High proliferative cancer cells have a high demand for fat and obtain energy by increasing the

absorption of exogenous lipids or by overactivating synthetic pathways. Both metabolite

utilization and metabolic pathway regulation, fatty acid metabolism in tumor and immune cells in TME play a crucial role in coordinating immunosuppression, providing opportunities for tumor to escape the immune defense mechanism of the body, and further promoting the development of GC.

### Toxic metabolites

In the TME, due to the complex metabolic increase caused by the participation of many cells, it shows the characteristics of increasing the concentration of metabolite lactic acid, kynurenine and K^+^, and the metabolic disorder of different cells. These metabolites are defined as toxic metabolites because they alter the pH of TME in such a tight resource race, and passivation of immune cell function and multiple pathways are conducive to the survival of tumor cells.

Lactic acid is commonly considered a toxic metabolite in glycolytic metabolism, which causes TME acidification and largely promotes malignant progression [112–114]. Tumor growth is supported by inducing new blood vessels and providing a substrate for the proliferation of cancer cells. Colegio et al. demonstrated that tumor cell-derived lactic acid polarizes macrophages and promotes tumor growth primarily by inducing the expression of vascular endothelial growth factor (VEGF) and arginase 1(Arg1) in macrophages primarily through HIF 1-a [35, 115]. Lactic acid in TME inhibits anti-tumor immune response by negatively regulating innate and adaptive tumor-infiltrating immune cells [28]. The researchers found that lactic acid can differentiate macrophages into M2-like states. In addition, targeted inhibition of LDHA inhibits the ability of macrophages in GC to differentiate into M2 phenomules [116]. DCs are thought to be the originators of immune responses, including those directed against tumors [117]. The researchers demonstrated that co-cultures of melanoma and prostate cancer multicellular tumor spheres (MCTS) produced little M-CSF and IL-6, but high lactate levels. In addition to lactic acid culture during the differentiation process of DCs, TADCs produced by DCs in vitro can be similar to those produced in MCTSs(monocytes invade MCTSs and differentiate into cells) of melanoma and prostate cancer. However, blocking lactic acid production in melanoma MCTS co-cultures restored the TADC phenotype to normal [118]. High levels of lactic acid significantly promoted the differentiation of monocyte derived DCs into TADCs, which showed reduced glucose consumption, up-regulation of mitochondrial respiration genes and inhibition of mTORC1 activity [119]. By loading lymphocytes from FoxP3YFP-CRE mice with a pH indicator dye and incubating them with lactic acid, the fluorescence display increased, indicating that T_reg_ cells absorb lactic acid as their own energy source. At the same time, the T_reg_ cells subsets given low and high glucose culture were purified and their function was evaluated. It was demonstrated that the high glucose environment inhibited the function of T_reg_ cells, but when the researchers added lactic acid equivalent to the TME to the environment, the function of T_reg_ cells was restored [120]. It has been shown that the TME rich in lactic acid promotes the function of T_reg_ cells and thus plays a tumor-promoting role. Alpaslan et al. reported that melanoma with high MCT1 expression can resist oxidative stress through uptake of lactic acid from the circulatory system, resulting in a stronger metastasis ability of the tumor [121]. In addition, the acid microenvironment caused by the accumulation of lactic acid has a protective effect on tumor cells [41]. So we suspect that treatment for GC based on lactic acid inhibition targets may work well.

Different concentrations of NO in TME and from different cell production sources showed dual anti-tumor or pro-tumor effects [122, 123]. For example, Bal-Price et al., described that low concentrations of NO promoted pheochromocytoma 12 (PC12) cell proliferation through cGMP, whereas high concentrations of NO inhibited tumor cell proliferation by inhibiting glycolysis and respiration [124]. In addition, NO produced by intestinal cells can reduce the inflammatory response of inflammatory colon cancer by reducing the infiltration of macrophages, while NO derived from immune cells can activate macrophages, aggravate the degree of inflammation and promote the occurrence of cancer [123].

K + is a double-edged sword for tumor immunotherapy. Cancer cells grow beyond their nutrient supply, and tumors often contain areas of necrosis. High levels of necrosis lead to a high concentration of K + accumulation in the TME [125]. T cells are believed to be able to protect the body from cancer and perform immune functions in immune organs and tissues [126]. Through the study of cancer patients who did not respond to immunotherapy, it was found that although there was a large amount of immune T cell infiltration around the tumor, these T cells were often not able to attack the cancer cells, but showed a state of exhaustion [125]. This is because the continuous accumulation of K + in TME will seriously affect the metabolism of T cells, triggering the restriction of their nutrient intake and functional energy, resulting in the inability of T cells to absorb nutrients from the external environment and lose the ability to attack cancer cells [127]. In addition, T cells in “starvation” are more likely to autophagy, and then release intracellular K + into TME, further increasing the concentration of K + in TME, which forms a vicious cycle. No matter how many T cells there are, they are just accomplices to further worsen the tumor [128]. On the other hand, the researchers also found that in TME with high concentrations of K^+^, the epigenetic modification of T cells changed, triggering stem cell-like characteristics of T cells. In this state, it is equivalent to setting a prohibition on the differentiation of T cells, resulting in stem cell-like T cells with strong self-replication ability and differentiation potential. Once the prohibition is lifted, they have the ability to kill tumors. It is difficult to turn on prohibitions at high concentrations of K^+^ in vivo, but it can be achieved with adoptive T cell immunotherapy. These stem cell state T cells are capable of differentiating into tumor killer cells and have achieved excellent tumor control in mice [128]. In conclusion, high concentrations of K can be used as a protocol for adoptive T cell immunotherapy. These “stem cell state” T cells are considered to be the most effective treatment for GC, suggesting that “stem cell state” T cells are expected to improve the efficiency of tumor immunotherapy, which will be a major advance in GC treatment. Nevertheless, high concentration of K will undoubtedly promote the progression of GC in vivo, so the control of K concentration is also crucial for the immunotherapy of GC.

**Fig. 2:**
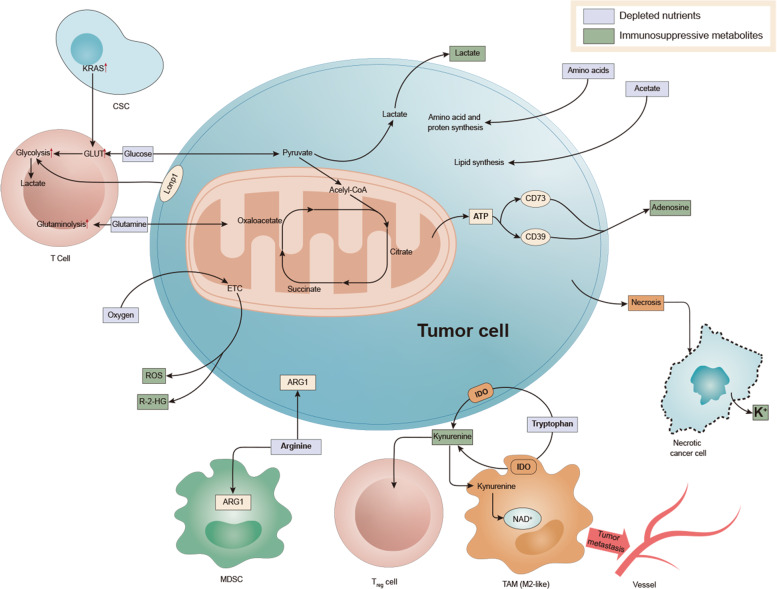
Associated immune metabolism in the tumor microenvironment. Enhanced glycolysis of tumor cells resulted in an increase of lactic acid in the microenvironment. Abnormal amino acid metabolism increased glutamine and canine urine. Abnormal fatty acid metabolism will lead to more dependence of immunosuppressive cells on FAO pathway. The above three metabolic abnormalities will make the function of immune cells abnormal and the transformation of immune phenotype, conducive to the growth of cancer cells.

To sum up, in the TME, due to the special metabolic mode of the tumor, a special microenvironment rich in high lactic acid, high concentration of K^+^ and NO is produced, which inhibits various immune cells and exerts a tumor-promoting effect (Fig. 2).

### Microenvironment beneath the microflora

The stomach was once thought to be a sterile environment, but it is now known to house many bacterial species [129]. The microbiome in the human stomach regulates many host cell life processes, including metabolism, inflammation, immunity, and cellular responses. It is becoming increasingly clear that microorganisms can also influence cancer development [130]. Once the microflora environment is maladjusted, it will lead to many diseases such as obesity, diabetes and tumor [131–133]. A study demonstrated that Sphingobium yanoikuyae was less abundant in the stomach of patients with GC than in patients with superficial gastritis, and that the bacteria were able to degrade aromatic hydrocarbons, a potentially carcinogenic molecule. Meanwhile, this study was the first to reveal a negative correlation between Sphingobium yanoikuyae and GC [134]. Compared with the normal control group, the abundance of lactic acid bacteria in GC patients increased [135]. Because both lactobacillus and lactobacillus are lactic acid producing microorganisms, and lactic acid can be used as an energy source for tumor growth and angiogenesis, theoretically helping tumor progression [136]. These facts all indicate that the occurrence of GC may be related to the imbalance of microorganisms in the gastrointestinal tract.

**Fig. 3:**
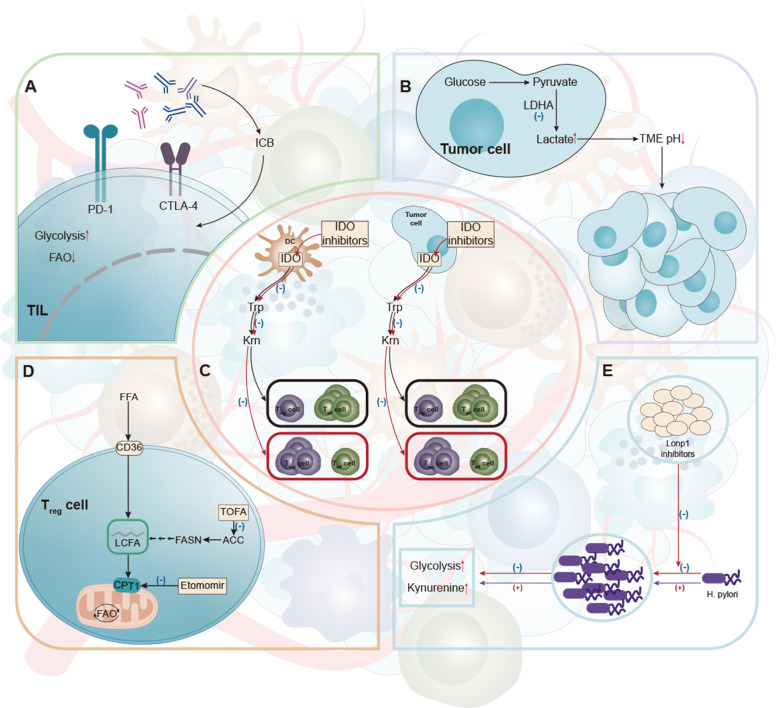
Therapies that targeting immune metabolism. **A** Increased checkpoint receptor expression is usually a result of low glucose, acidity, or lactic acid in the TME, and the involvement of these receptors leads to immunosuppressive phenotypic characteristics of reduced glycolysis and increased FAO. Antibodies against these checkpoint receptors have successfully restored glycolysis, which in turn supports the function of antitumor effectors within immune cells. **B** LDHA is required for the conversion of pyruvate to lactic acid and is associated with the development, maintenance, and metastasis of cancer. LDHA is a potential therapeutic target. **C** Inhibitory immune cells, including Treg, TADCs and MDSC, increased the expression of IDO, an enzyme responsible for metabolizing tryptophan into canine urine. IDO-targeting activity inhibits these suppressive immune cells in the tumor. **D** Immunosuppressive Treg cells often use FAO as a way to produce energy. The metabolism of Treg can be improved by using ACC inhibitors, CPT1 inhibitors or CD36 inhibitors, providing potential targets for the treatment of gastric cancer. **E** In the TME of specific gastric cancer, the microflora was changed. In gastric cancer patients, a large increase of H. pylori led to an increase in tumor cell glycolysis and Lonp1. Downregulation of Lonp1 expression significantly reduced glycolytic metabolic transformation and gastric cell proliferation associated with low diversity of H. pylori infection.

Increased glycolysis [43] and Lon protease 1 (Lonp1) were shown in H. pylori infected gastric epithelial cells. We found that downregulation of Lonp1 expression significantly reduced glycolytic metabolic transition and gastric cell proliferation associated with low diversity of H. pylori infection. In addition, Lonp1 overexpression in gastric epithelial cells also promoted glycolysis and cell overgrowth, suggesting that the effect of H. pylori is dependent on Lonp1 [137] (Fig. 3).

Unlike other types of cancer, the presence of H. pylori in GC affects the regulation of amino acids in the TME. In a clinical study, the presence of H. pylori in both positive and negative colorectal cancer patients showed reduced plasma tryptophan levels and increased kynurenine levels [138]. Arginine plays an important role in the immune process of macrophages against pathogens [62]. NO produced by inducible nitric oxide synthase (iNOS) can play an antibacterial role [139]. Using mouse gastric macrophages, H. pylori up-regulates the production of arginase II (Arg2) to consume l-arginine and produce spermine, while reducing the production of NO by reducing the translation of iNOS in macrophages [140]. These behavioral signs suggest that H. pylori may promote cancer development by allowing immunosuppression and immune escape.

In the development process of chronic atrophic gastritis to enteric GC, the gastric juice PH in the stomach will gradually increase, so the number of H. pylori in the stomach will gradually decrease, and the increase of other bacteria besides H. pylori in the stomach suggests that other bacteria in the stomach are also closely related to the occurrence of GC [141]. Recently, Ling et al. investigated the relationship between gastric mucosal microbiota and immunosuppressive cells in TME in 64 patients with GC who did not receive preoperative chemotherapy. It was found that some bacteria in TME, such as Stenotrophomonas and Selenomonas, were positively correlated with BDCA2 + Plasmacytoid Dendritic cells (pDCs) and Foxp3+T_reg_, which play immunosuppressive effect in GC, respectively. Comamonas and Gaiella were negatively correlated with BDCA2 + pDCs and Foxp3+ T_reg_, respectively. Gastric mucosal microorganisms may promote the occurrence and development of GC by regulating the increase of BDCA2 + pDCs and Foxp3+ T_reg_, leading to immune escape, which may provide a basis for the establishment of a new anti-tumor therapy strategy targeting gastric microorganisms [142].

In conclusion, the imbalance of bacterial community, especially H. pylori, plays a pivotal role in the occurrence of GC. Infection with H. pylori will increase glycolysis, affect amino acid metabolism, and increase toxic metabolites. These changes largely promote the progress of GC.

## Therapy

### Targeting glucose and lactic acid

Immune checkpoint blockade has emerged as a promising immunotargeting strategy that has been clinically approved for using in a variety of cancer types. Immune checkpoint signaling can regulate metabolic activity. For example, the expression of programmed cell death 1 ligand 1 (PD-L1) in cancer cells can drive the activation of Akt-mTOR and glycolysis in cancer cells, increase glucose uptake, and enhance the ability to compete with T cells for glucose [143]. Increased checkpoint receptor expression is usually the result of low glucose, acid, or lactic acid in the TME. Immuno-checkpoints including PD-1, PD-L1 and CTLA-4, function to some extent by inhibiting glycolycolysis while increasing lipid solution and FAO. Although the use of checkpoint lockdown has significantly improved the effectiveness of cancer treatment, it must be noted that current strategies are most effective against highly glycolytic tumors (Fig. 3) [144, 145]. Research on GC immunotherapy by Kohei Shitara et al. can show that PD-L1 expression alone is not sufficient to screen people who can benefit fully from immunotherapy, and new efficacy-related markers must be sought to help further optimize patient selection for GC patients. The markers of the candidates include tumor mutation load, microsatellites, ctDNA, GC molecular parting, etc. However, the correlation between these markers and immunotherapy effect in advanced GC has not been fully demonstrated and needs to be further explored. Beyond that microRNAs (miRs) are also take part in the development of GC. MiRs regulate gene expression by inhibiting mRNA translation or promoting its degradation. Samples of GC tissue were taken from patients with GC for miR-140 expression testing. MiR-140 expression significantly decreased in H. pylori-positive GC, while PD-L1 expression increased significantly in H. pylori-positive GC. These findings suggest that miR-140 has an anti-GC effect by targeting the immunoassay molecule PD-L1. Therefore, miR-140 may be a promising and new immunotherapy target for GC treatment [146].

We also learned that tumors are classified as “immune cold tumors” and “immune hot tumors,” in which an active immune response is taking place. Therefore, we named these tumors “immune hot tumors.” Instead, there are “immune cold tumors.” Different immune characteristics are also different for treatment. Sato Y et al. showed experimentally that in immune thermal tumors, some patients with thermal tumor gene subtypes are recommended to receive anti-PD-1 therapy prior to the currently approved treatment time [147]. The clinical analysis results of GC samples showed that the genes RP11-1094M14.8 and CXCL9 were expressed more in hot tumors, and CXCL9 was positively correlated with the patient’s prognosis. Since the two genes are also related, knocking out the RP11-1094M14.8 gene significantly reduces the expression of CXCL9. To this end, we venture to speculate that the expression of these two genes in GC patients may be related to the good prognosis of GC [148]. And other researchers divided GC patients into two categories, hot tumors and cold tumor patients. Claudine-3 (CLDN3) gene was found to have high expression in cold tumors, and it was associated with a reduction in CD8-T cells in the GC by inhibiting the expression of MHC-I and CXCL9 [149]. Therefore, for different pathological types of GC cells, it is also important to see if they are one of the hot and cold tumors.

The Warburg effect mentioned above is based on the up-regulation of glycolytic enzymes such as lactate dehydrogenase A (LDHA), which is necessary for the conversion of pyruvate to lactic acid. Mitochondrial venom I (TOP1MT) is associated with the development, maintenance, and metastasis of cancer. The lack of TOP1MT inhibits aerobic oxidation of glucose, but enhances glycoentic enzymes in normal cells. Researchers found that TOP1MT expression was lower in GC samples than in adjacent normal tissues. Knocking out the TOP1MT gene significantly promotes GC migration and in vitro and in vivo invasion. In addition, the above effects of TOP1MT were associated with a significant increase in expression of LDHA [150]. Then by inhibiting LDHA, the expression of the TOP1MT gene can be reduced, thereby reducing the possibility of GC transfer. LDHA levels are associated with the size and clinical stage of GC [112, 151]. Yao et al. found that LDHA plays an important role in the development and development of Esophageal Squamous Cell Carcinoma (ESCC) by regulating cell growth. LDHA may be a potential therapeutic target for ESCC (Fig. 3) [152]. Therefore, we speculated whether inhibition of LDHA, an enzyme associated with lactate metabolism, would have a similar effect on gastric tumors.

### Targeting amino acids

Regulating amino acid levels is an important therapeutic entry point. Due to the multi-pathway carcinogenic effects of IDO1 and kynurenine, a variety of IDO inhibitors have been evaluated in clinical trials. Targeting IDO inhibitors and multi-pathway kynurenine failure can promote the therapy of GC [153]. A clinical trial using the IDO1 inhibitor Epacadosta and gene disruption blocking IDO1 activity in colorectal cancer cells has demonstrated that IDO1 inhibition enhances the effects of radiation therapy for colorectal cancer, and provides basic principles and mechanism insights for the study of IDO1 inhibitors as adjunct therapy to radiotherapy in patients with locally advanced sporadic and colitis associated colorectal cancer (Fig. 3) [154]. IDO1 is positively correlated with six collagen genes. Knockdown IDO1 reduces the expression of LOXL2, COL6A1, COL6A2 and COL12A1 in GC cells. Among them, reduction of COL12A1 inhibited cell migration more significantly than reduction of other cells. The researchers established a stasi model of epidemic lymph node location and found that IDO1 and COL12A1 synergistically promote GC metastasis. So we speculate that IDO1 and COL12A1 are both promising targets for GC anticancer therapy [84].

Arginine and glutamine support therapy has shown good effects for a variety of severe diseases with high consumption, such as trauma, hemorrhagic shoc, tumor and acute inflammation [155, 156]. Different arginine-related treatments can be administered to different metabolic types of tumors. For tumors that cannot independently synthesize arginine, arginine depletion is a research direction that can be carried out [157, 158]. A Randomized Clinical Trial was conducted to investigate the lowering of arginine--the arginine-lowering agent pegylated arginine deiminase (ADI-PEG20) arginine deprivation can prolong the survival of patients with advanced ASS1-deficient malignant pleural mesothelioma [159]. A recent study also showed that TINCR mediated regulation of PADI1-MAPK-MMP2/9 signaling pathway plays a key role in nasal pharyngeal cancer (NPC) progression and chemical resistance, suggesting that TINCR may be a therapeutic target for GC [160]. IRX1 is a tumor suppressor gene of GC. The researchers identified the protein arginine methyltransferase 5 (PRMT5) as the primary upstream regulator of IRX1, used to determine GC progression. PRMT5 expression was significantly increased in human GC tissues (433 of 602, 71.93%) compared with normal gastric mucosa and showed diagnostic and prognostic potential. In general, PRMT5 promotes tumor growth and metastasis of GC cells by silencing IRX1.Targeting PRMT5 in GC may inhibit the malignant characteristics of GC and have new therapeutic potential [161]. ASCT2 and glutamine synthetase (GS) were expressed in GC cells or human GC samples using molecular biological methods. The results showed that Gln mediated GC growth, and the therapeutic effect of GLN-targeted therapy depended on the unique expression patterns of ASCT2 and GS in specific GC groups [162]. Under the condition of the use of glutamine antagonists, respectively observed the metabolism of cancer cells and T_eff_ cells, decrease oxidation and glucose metabolism in the cancer cells result in hypoxia, acidosis, reduce and nutrient consumption in cancer cell, but T_eff_ cells by significantly raised oxidative metabolism and longevity, highly activated phenotype is used to react to glutamine antagonism effect [163]. In contrast, the use of glutamine antagonism in the TME relatively improves the activity of anti-tumor cells. In a mouse model of acute myeloid leukemia (AML), blocking glutamine metabolism with a glutaminase inhibitor (CB-839) significantly impaired antioxidant glutathione production in multiple types of AML, leading to an increase in mitochondrial reactive oxygen species (mitoROS) and apoptosis. In addition, glutaminase inhibition sensitizes AML cells to adjuvant agents that further disrupt the mitochondrial redox state, such as arsenic trioxide (ATO) and tetrochartonine (HHT) [164]. This suggests that the combination of drugs that target glutamine metabolism and interfere with mitochondrial redox state represents an effective and potentially widely applicable therapeutic strategy for multiple types of leukemia [164].

### Targeting lipid metabolism

Highly proliferating cancer cells exhibit a strong lipid affinity [165], and fatty acid accumulation has a significant impact on immune cells in the TME. Several drugs targeting lipid metabolism have been studied for the treatment of GC. Inhibition of key enzymes involved in lipid metabolism is one of the important strategies for cancer treatment.

One way to inhibit key enzymes involved in lipid metabolism is to promote anti-tumor immunity by enhancing the effects of CD8^+^T cells. For CD8^+^T cells, cholesterol can promote T cell receptor(TCR) aggregation during activation and increase the formation of immune synapses by maintaining cell membrane fluidity [166, 167]. Inhibition of Cholesterol Acyltransferases1 (ACAT1) increases cholesterol in CD8^+^T cell membranes. Therefore, ACAT1-deficient CD8^+^T cells have been shown to enhance the function and proliferation of effector cells by accelerating immune synapse formation and improving TCR aggregation and signal transduction to increase the damage to tumor cells and improve the therapeutic effect of GC [168]. At the same time, ACAT1 inhibitor avamoib combined with anti-PD-1 immunotherapy can significantly improve the survival rate of tumor-bearing mice [168]. Therefore, the key metabolic regulator ACAT1 may be a potential target for immunotherapy of GC.

Targeting Fatty acid synthase (FAS) or FAO is also beneficial for mouse tumor models. Inhibition of FAS by acetyl-coa carboxylase (ACC) inhibitor TOFA restores normal levels in DCs and significantly enhances the efficacy of GC vaccine [169]. FAO blockade can inhibit the function of immunosuppressive cells including M2-like macrophages and T_reg_ cells [109, 170] The high expression of CPT1A is closely related to grade, LN metastasis, clinical stage, and poor prognosis of GC patients. The overexpression of CPT1A activates FAO of GC cells by increasing NADP^+^/NADPH ratio, and promotes tumor progression of GC cells. The FAO inhibitor Etomoxir was used to inhibit the growth and migration of CPT1A overexpressed AGS/BGC823 cells. It was found that Etomoxir reduced FAO and NADPHNADP^+^/NADPH ratio, and inhibited the growth and migration of CPT1A overexpressed GC cells. This supports that CPT1A may be a new prognostic and potential therapeutic target for patients with GC [171].

Fatty acid transporter CD36 is a very specific target of T_reg_ cells. Compared with T_reg_ cells from other tissues and circulatory system, T_reg_ cells in tumor have higher fatty acid absorption capacity and high expression of CD36 [172, 173]. CD36 knockout of T_reg_ cells does not induce autoimmunity, but enhances antitumor activity.CD36 mediates palmitic acid-induced metastasis of GC through the AKT/GSK-3β/β-catenin pathway [174]. Anti-CD36 monoclonal antibody promoted the apoptosis of tumor T_reg_ cells in mouse tumor model, and led to a significant increase in tumor infiltration of CD8^+^ T cells and had a synergistic effect with PD-1 antibody. CD36 molecules on the surface of tumor infiltrating CD8^+^ T cells can absorb oxidizing low density lipoprotein (OxLDL), activate lipid peroxidation, and inhibit the effector function of CD8^+^ T cells [175]. Targeting CD36 in T_reg_ cells can inhibit the polarization of M2-like macrophages [108], and may also supplement the blocking effect of PD-1 in treatment to offset the failure of tumor-infiltrating T cells [176], and reprogram TME, which may become a potential therapeutic target for clinical intervention in GC [173].

At present, nanoparticle-mediated T cells play an important immune role in lipid metabolism reprogramming in TME. The researchers found that aCD3/F/ANs can promote the proliferation of T cells in the TME with low glucose level by enhancing fatty acid metabolism, and enhance its anti-tumor effect. Anti-cd3e F (AB’)2 fragment modification of fenofibrate-coated amphiphilic nanoparticles can reprogram the mitochondrial lipid metabolism of T cells to make use of different energy substrates, which may be a feasible strategy to improve immunotherapy [177].

KRAS mutations have been reported in many types of cancer, including pancreatic, lung, colon, breast, and GC [178]. Studies have shown that KRAS promotes adipogenesis, and this activation leads to different lipid profiles. Gene expression analysis showed that KRAS was related to adipogenic gene characteristics and specific induction of fatty acid synthase (FASN). KRAS is involved in the induction of FASN and the promotion of adipogenesis. Therefore, FASN may be a unique target for KRAS-associated GC [179].

### Effects of gastrointestinal microbiome

The occurrence and development of GC is closely related to the disorder of intestinal microflora. Immunotherapy has made an important breakthrough in the treatment of GC. Nivolumab is used in the treatment of unresectable advanced or recurrent GC that progresses after chemotherapy [180]. More and more studies have shown that intestinal microbial ecology is also one of the important factors affecting the effect of cancer immunotherapy. Specifically, in the study of patients treated with antibiotics within 2 months before and after the use of nivolumab, the effect of immunotherapy was affected due to the large number of intestinal microbes killed by antibiotics, and the overall survival of patients was significantly reduced [181]. Different types of gut microbes can also have different effects on the effects of immunotherapy. Patients with high levels of clostridia bacteria and Faecalibacterium in their gut are more likely to respond to treatment with PD-1 antibodies, those with more bacteroidetes in their gut were less likely to respond to treatment [181, 182]. Although approximately 60% of GC patients did not respond to navuliumab as Posterior Line therapy, progressive disease (PD) patients who responded to navuliumab therapy had a more diverse gut microbiome compared to non-PD patients. Recent studies have also provided clues about the effects of gut microbes on immunotherapy for advanced GC. This study was recently presented at the American Society of Clinical Oncology gastrointestinal Oncology Symposium 2021 by Dr. Yu Sunakawa. They have found that the upregulation of bacterial invasion of epithelial cell pathways in Kyoto Encyclopedia of Genes and Genomes (KEGG) is related to the efficacy of patients receiving navuliumab treatment. Although after correction by Bonferroni correction, no obvious association was found between these pathways and advanced GC, it still provides an idea for us to find that bacterial invasion of epithelial cell pathways may serve as a potential prognostic marker for the efficacy of navuliumab in advanced GC. Odoribacter and Veillonella were associated with the efficacy of navulizumab through exploration and analysis. It is suggested that the detection of GC specific intestinal microflora may predict the efficacy of immune checkpoint (ICB) [183].

## Conclusion

Since the approval of ICB, tumor immunotherapy has become a hot spot in cancer therapy, and Tumor immunologic drugs have begun to find their way. Advances have been made in combination immunotherapy combining ICB and tyrosine kinase inhibitors (TKI). For example, in the KEYNOTE-426 trial, when combined with pembrolizumab and vascular endothelial growth factor receptor (VEGFR) inhibitor axitinib, compared with monotherapy for primary advanced/metastatic renal cell carcinoma (mRCC), it improved progression-free survival (PFS) and overall survival (OS) in mRCC patients [184]. This traditional combined ICB development method is also applicable in the treatment of GC. Neoantigen-based personalized tumor immunity is also a new type of tumor immunotherapy.

In this review, enzymatic changes in various metabolic pathways of gastric tumor cells lead to changes in glycolysis, amino acid and protein synthesis and lipid biosynthesis pathways, leading to metabolic disorders and the formation of TME, resulting in local immunosuppression of tumor infiltrating immune cells. In addition, the regulation of tumor metabolites and metabolic pathways can cause tumors to escape the body’s immune defenses. Therefore, future studies should explore and identify targets that can inhibit or change GC metabolism to enhance the availability of nutrients in TME or regulate immune metabolism. Selectively targeting metabolic markers specific to GC cells can help improve the curative effect of GC treatment and develop new effective anti-GC drugs.

Focusing on gastric microecology, H.pylori interacts with the inherent gastric microflora, and the changes of gastric microflora may be used as markers to predict the progression of GC. The study of insulin-gastrin (INS-GAS) transgenic mouse model suggests that further study is needed on the role of immune regulation in preventing the progression of gastric diseases. In the future, large-scale and prospective studies should be conducted to identify the specific microorganisms associated with GC and the changes in gastric microecology during the progression of GC, which provides another idea for the treatment of GC.

In order to optimize the efficacy of immunotherapy, we also need to explore the combination of immunotherapy and immunotherapy, the combination of immunotherapy and targeted therapy, and the combination of immunotherapy and chemotherapy, which are also the focus and direction of immunotherapy research in GC [185].

## Data Availability

Not applicable.
